# The association of prescriber prominence in a shared-patient physician network with their patients receipt of and transitions between risky drug combinations

**DOI:** 10.1007/s41109-025-00721-y

**Published:** 2025-07-21

**Authors:** A. James O’Malley, Ellen Meara, Nancy E. Morden, Erika L. Moen, Xin Ran

**Affiliations:** 1 Department of Biomedical Data Science, Geisel School of Medicine at Dartmouth, Lebanon, NH 03756 USA; 2 The Dartmouth Institute for Health Policy and Clinical Practice, Geisel School of Medicine at Dartmouth, Lebanon, NH 03756 USA; 3 Department of Computer Science, Dartmouth College, Hanover, NH 03755 USA; 4 Department of Health Policy and Management, Harvard T.H. Chan School of Public Health, Boston, MA 02115 USA; 5 National Bureau of Economic Research, Cambridge, MA 02139 USA; 6 United HealthCare, Minnetonka, MN 55343 USA

**Keywords:** Polypharmacy, Physician shared-patient network, Network centrality, State-space, Transition matrix, Risky prescribing, Deprescribing

## Abstract

We are generally interested in the association between a prescribing physician’s position in a physician shared-patient network and their patients’ receipt of risky drug combinations. An informal physician network (not restricted to a hospital or a health system) of physicians based in Ohio was constructed based on overlapping care of patients between physicians reflected in face-to-face visits in Fee-for-service Medicare claims for Ohio-residing beneficiaries. Separately, Medicare prescription drug events for beneficiaries receiving opioids, benzodiazepines, or non-benzodiazepine sedative hypnotics (sedative hypnotics) prescribed by these physicians in 2014 were used to map patients’ drug status with respect to these three classes. We assigned patient prescription receipt to time-varying drug states and linked each drug state transition to a “responsible” prescribing physician. Outcomes of interest include transitions across drug states, particularly those resulting in combinations of increased risk (e.g., a benzodiazepine or sedative hypnotic with an opioid), and patients’ time to discontinuation of overlapping prescriptions of an opioid, benzodiazepine, and a sedative hypnotic while the key predictors of these transitions reflected characteristics of a prescriber’s physician network position and physician speciality. We found that among beneficiaries receiving none of the three risky drug groups, patients seeing physicians with higher closeness centrality (shorter average path lengths to other physicians through the network) were less likely to transition to two or three risky drugs; and they were more likely to discontinue overlapping prescriptions of an opioid, benzodiazepine, and sedative hypnotic. Compared to PCPs, psychiatrists appeared more likely to prescribe risky drug combinations, and their patients were less likely to discontinue overlapping three-drug prescriptions. This work demonstrates that characterizing physicians’ prescribing behavior in relation to their position in shared-patient networks may reveal strategies for optimizing network-based interventions to improve prescribing quality.

**Supplementary Information:**

The online version contains supplementary material available at 10.1007/s41109-025-00721-y.

## Introduction

High-risk or “risky” prescribing is a pressing public health concern. Risky prescribing include inappropriate use of drugs with significant risks of adverse effects, multiple medication use (polypharmacy), or prescription use that deviates from guidelines developed for safety; these are all of particular concern among older adults who generally are more susceptible to adverse drug effects than younger adults (Dreischulte and Guthrie 2012; Gnjidic et al. 2013; Bushardt et al. 2008). Although adults aged 65 years and older comprise less than 15% of the U.S. population, this group consumes one-third of prescription medications and 40% of nonprescription medications (Fulton and Riley Allen 2005; Werder and Preskorn 2003). Especially alarming is the common, concurrent use of opioids and benzodiazepines, and of opioids and non-benzodiazepine sedative-hypnotics (sedative-hypnotics), among older adults (Musich et al. 2020; Rhee 2019; Maree et al. 2016; Pisani et al. 2009; Schepis et al. 2019; Dublin et al. 2011). Older adults metabolize drugs more slowly, and the aging brain becomes more sensitive to drug effects, making older adults more vulnerable to adverse events attributed to medication combinations (Noble 2003; Corsonello et al. 2010; Frazier 2005; Riker and Setter 2012; Ziere et al. 2006). Concurrent use of opioids and benzodiazepines and of opioids and sedative-hypnotics can lead to additive side effects including sedation, loss of balance, and confusion, and such drug combinations are associated with respiratory depression (Musich et al. 2020; Corsonello et al. 2010; Cho et al. 2020; Dowell et al. 2016; Sun et al. 2017).

Given the prevalence and health impact of risky prescribing, especially among older adults (involving opioids, benzodiazepines, and sedative-hypnotics), we sought to identify predictors of physician prescribing of these risky drugs in order to identify potential levers to reduce such prescribing. Past work regarding physician shared-patient network characteristics and patient outcomes raised the question of whether network position characteristics (such as the number of other physicians with whom they shared patients, known as “degree,” and their prominence in the network as measured by any of the many centrality measures including a measure of their average path length to other physicians through the network, known as “closeness” centrality), are associated with patients” risky drug receipt (Moen et al. 2019; Geissler et al. 2020; Barnett et al. 2012; Moen et al. 2016, 2018). Social network analysis is a valuable tool to study physician collaboration and health systems in general. Physician networks in which edges between two physicians are defined based on the extent of their observed clinical encounters with the same patients, provide a new lens through which to study variability in the health system. In general, “shared-patient networks” have been associated with patient care in studies of the adoption of new medical technology, healthcare spending, and utilization of services (Barnett et al. 2012; Moen et al. 2016, 2018; Landon et al. 2018; Pollack et al. 2013). Having more shared-patients with other physicians was found to be associated with a reduction in risky drug prescribing (Fattore et al. 2009; Arnold et al. 2021; Ong et al. 2016). However, the impact of other characteristics of physicians’ network position (e.g., degree, closeness, and other centrality measures) on their prescribing behavior is less studied. Understanding how network position relates to prescribing decisions is clinically important because the decision to prescribe a potentially risky drug combination by one physician may impact the prescribing decisions of other physicians regarding risky drugs. If physicians with more (less) prominence in the network are associated with higher likelihood of risky prescribing compared to physicians on the outskirts or “periphery” of the network, physician interventions aimed at reducing risky prescribing could exploit physician-network effects by targeting the most (least) prominent physicians.

Herein, we study the association between physician network position and risky prescription drug received (specifically fills for opioids, benzodiazepines and sedative-hypnotics singly and in combination) among their patients. We model patients’ prescription receipts as prescription states and evaluate whether concerning transitions are predicted by physician prominence in shared-patient networks, other characteristics of physician network position and physician specialty characteristics. While there are many measures of prominence, we use four classic centrality measures to capture different aspects of physicians’ relative prominence in a network designed to reflect physician collaboration and communication (Freeman 1978; Bonacich 1972). Other network measures considered (see Section 2.5) include clustering coefficient, or the extent that the physicians that a given physician is directly connected to (“peers”) are interconnected in the sense that they share patients with each other (Barrat et al. 2004).

To our knowledge, a study of the association between physicians’ prominence in a network and physicians’ risky drug prescribing has not previously been undertaken. This paper extensively extends our prior work (O’Malley et al. 2024) published in the Proceedings of the Complex Networks 2024 Conference, which is focused on the addition of drug categories from an initial state of zero drugs; the current paper considers multiple other transition types in addition to those from the baseline zero state and, therefore, is a more comprehensive treatment of this topic. We believe that the results of the research reported herein may ultimately inform the prioritization and development of shared-patient-network-based interventions to reduce risky prescribing in the U.S. healthcare system.

## Methods

### Overview of data and subjects

Among a 40% random sample of Medicare beneficiaries, we used Part B and outpatient files in 2014 for beneficiaries residing in Ohio to form a list of physician-patient encounters from which a physician shared-patient network, which yielded the network position predictor variables for our analyses, was developed. Ohio was selected for this study because it has a large population with a high incidence of adverse events related to opioids, and Ohio had the fifth-highest overdose death rate in the U.S. in 2014 according to the CDC (Centers for Disease Control and Prevention 2014a, b). Furthermore, Ohio has a relatively large amount of diversity along multiple dimensions (e.g., urban versus rural, by race) and had a large enough population of physicians and patients to allow meaningful effect sizes to be detected with reasonable levels of statistical power. As such, Ohio is both a middle ground between many other states (thereby offering some relevance to almost any other state) and is practical to analyze. As noted in the Discussion, we do not want to imply that Ohio is representative of all other states and emphasize that our study is intended to be symbolic of analyses that may be performed on other states and nationally.

Separately, to define beneficiary cohorts and outcomes, we extracted the 2014 Medicare Part D prescription drug event file for beneficiaries who had received prescriptions in one of three groups of potentially risky drugs: opioid analgesics (opioids), benzodiazepines, and non-benzodiazepine sedative-hypnotics (sedative-hypnotics) prescribed by physicians in the Ohio-based shared-patient network. Although the physician shared-patient network was limited to Ohio, outcome analyses involved all beneficiaries linked to physicians in the network, including those residing outside of Ohio; the study cohort definition is further described in Fig. A1 of the Supplemental Appendix. Physician specialty assignment was retrieved from the National Plan and Provider Enumeration System (NPPES) (Centers for Medicare and Medicaid Services 2012), and advanced care practitioners including physician assistants and nurse practitioners were excluded from the study.

### Patient prescription states

We used the observed prescription fill events of each patient in the study cohort to generate discrete time intervals of their drug exposure capturing the start and end of each prescription. Each time a patient received a new drug in one of the three groups, they were assigned to one of the $2^3=8$ possible combinations of prescription states involving opioids, benzodiazepines, and sedative-hypnotics until they received another prescription, or their fill (days supply) ended (see Supplemental Appendix for details). State *zero* indicates receiving none of the drugs in these three groups. States $\{O, B, S\}$ denote the three singleton drug prescription fills while states $\{OB, OS, BS\}$ denote overlapping prescriptions filled for opioid/benzodiazepine, opioid/sedative-hypnotic, or benzodiazepine/sedative-hypnotic, respectively. Finally, state *OBS* indicates overlapping prescription fills for an opioid, a benzodiazepine, and a sedative-hypnotic.

### Patient prescription state transition matrix

Using the eight possible prescription states defined above, we generated a time-ordered sequence of each patient’s prescription fill states across the study period. We then summed the prescription state transitions observed across all the patients in the cohort to form an 8 by 8 transition matrix $\textbf{M}$. Let *k* denote the patient, *t* the time-interval, *i* and *j* prescription states, and *D* the patient’s prescription state. Across all patients and time-intervals, the number of transitions from state *i* (the row) to state *j* (the column) is given by,

1 $$\begin{aligned} C_{i,j} = \sum _{k} \sum _{t} \textbf{1}(D_{kt} = i, D_{k(t+1)} = j), \end{aligned}$$

where $\textbf{1}(event)=1$ if the event is true and 0 otherwise, and the 8 by 8 transition matrix by,

2 $$\begin{aligned} \textbf{M} = \begin{bmatrix} \pi _{1,1} & \pi _{1,2} &... & \pi _{1,j} &... & \pi _{1,8} \\ \vdots & \vdots & \ddots & \vdots & \ddots & \vdots \\ \pi _{i,1} & \pi _{i,2} &... & \pi _{i,j} &... & \pi _{i,8} \\ \vdots & \vdots & \ddots & \vdots & \ddots & \vdots \\ \pi _{8,1} & \pi _{8,2} &... & \pi _{8,j} &... & \pi _{8,8} \end{bmatrix}, \end{aligned}$$

where $\pi _{ij}=C_{i,j}/(\Sigma _{j}C_{i,j})$ is the estimated transition probability from state *i* to state *j*, and that $\Sigma _{j}\pi _{i,j} = 1$.

### Physician responsible for patient prescription state transitions

To study the association between the transition that patients undergo and the network positions of their prescribers, we assigned each transition and associated time-interval to the physician considered responsible for the patient. When a drug is added, the responsible physician is the physician who prescribed it. However, when a patient appears to terminate a drug by failing to refill a prescription, no insurance claim is triggered. Thus, the identification of the responsible physician for ceasing a patient’s drug regime is challenging. For this reason, we limit the study of discontinuation to modeling the time until a patient fails to refill a drug or a combination of drugs and use the most recent prescribing physician as the physician responsible for discontinuation.

### Physician shared-patient network and summary positional measures

We built an undirected-weighted physician network for Ohio using all physician-patient encounters in the fee-for-service Medicare data for beneficiaries residing in Ohio in 2014. The nodes in the network represent physicians, and the edge weights reflect the intensity of patient-sharing between them. When enumerating the shared-patient network, we approximated a restriction that patient encounters be for the same episode of care by requiring that a patient had to have visited two physicians consecutively within 30 days in order to contribute to their edge weight; while some encounters within the same episode could be more than 30 days apart, we felt that the physicians with the strongest professional relationships would likely have many shared patients and so would still be captured even if some episodes were missed and that this was preferable to including edges that existed only due to a patient only visiting the physicians involved in an edge separately in distinct unrelated episodes of care (O’Malley et al. 2023). The final edge weight between two physicians is the number of patient encounters with both of those physicians within 30 days.

We restricted the network to the subgroup of physicians for whom there is a path through the network to every other physician (termed the largest connected component, denoted LCC) so that network-distance-based measures such as closeness centrality were well-defined; otherwise, nodes in different sub-networks with no paths to physicians in other sub-networks have undefined distances between them. The physician network positional measures for physicians in the LCC of the network were evaluated on the network formed across all of 2014; these are described below with mathematical definitions in the Supplemental Appendix.

Node strength: the total number of patients a physician shared with other physicians in the shared-patient network, reflecting the responsible physician’s tendency to make professional connections and their recognition by peer physicians.

Node degree: the number of distinct physicians in the shared-patient network with whom a physician shared patients.

Closeness centrality: a measure of the average shortest path distance a node is to all other nodes in the network (Freeman 1978), reflecting the potential for the responsible physician to efficiently transmit information to all other physicians in the shared-patient network. In a weighted network, shortest paths are measured in terms of inverse edge weights; more shared patients between two physicians leads to a shorter path between them and likely increased closeness centrality.

Betweenness centrality: the proportion of shortest paths between all pairs of other nodes in the network that intersect the given node (Freeman 1978), reflecting the responsible physician’s importance in terms of their ability to intersect information transfer in the network.

Eigenvector centrality: the extent to which a node is connected to central nodes in the network (Bonacich 1972), reflecting the responsible physician’s prominence when quantified by the prominence of their peer physicians. A physician with few connections who is connected to highly influential physicians may have high eigenvector centrality.

Local clustering coefficient: A measure of the extent that a physician’s peer physicians are inter-connected, reflecting the responsible physician’s inclusion in a tightly connected group of physicians and the phenomenon that “a friend of a friend is a friend” (Barrat et al. 2004).

### Statistical models and analyses

#### Modeling prescription state transitions resulting in increased risky combinations

In our first set of analyses, we reduce the aforementioned eight prescription states in Section 2.2 to four categories corresponding to overlapping fills of zero, one, two, and three distinct drug groups. State *zero*, filling zero drugs from the three drug groups, is the baseline state for every patient. States $\{O, B, S\}$ correspond to filling one distinct group of drugs. States $\{OB, OS, BS\}$ correspond to filling two distinct groups of drugs. State *OBS* is filling three distinct groups of drugs. For each of the four starting states, there are three possible ending states; for example, the transition from state *zero* to other states involves the addition of one, two, or three drug groups.

We used a multinomial mixed-effect regression model to examine the association of the responsible physician’s network position with the odds of transitioning to each different number of distinct drug groups. Suppose $Y_{kt}$ denotes the number of distinct drug groups in which patient *k* had received drugs in time interval *t* after an encounter with a physician and *u*, *v*, and *w* denote numbers of distinct drug groups. The odds of a change from *u* to *v* over that from *u* to *w* is modeled as,

3 $$ \begin{aligned} \log \left( {\frac{{\Pr (Y_{{kt}} = v)|Y_{{k(t - 1)}} = u}}{{\Pr (Y_{{kt}} = w)|Y_{{k(t - 1)}} = u}}} \right) = & \beta _{0}^{{(v)}} + \beta _{1}^{{(v)}} Patient_{k} \\ & + \beta _{2}^{{(v)}} Network_{{Phys_{{kt}} }} + \beta _{3}^{{(v)}} Volume_{{Phys_{{kt}} }} \\ & + \beta _{4}^{{(v)}} Specialty_{{Phys_{{kt}} }} + \theta _{k}^{{(v)}} + \gamma _{{Phys_{{kt}} }}^{{(v)}}, \\ \end{aligned} $$

where $\Sigma _{i=1:3}Pr(Y_{kt}=v_{i}|Y_{k(t-1)}=u)=1$ with $\{v_1, v_2, v_3\}$ $\ne u$ ensures that the probabilities of the three possible transitions from state *u* sum to 1. When $u=0$ and $u=3$ the three possible transitions are all prescribing or deprescribing events, respectively.

In Eq. 3, $Patient_k$ denotes the demographic characteristics for patient *k* including age, gender, and race and ethnicity; $Phys_{kt}$ is the physician who was responsible for patient *k*’s transition between time intervals $t-1$ and *t*, reflecting a change in the drug groups prescribed to them; $Network_{{Phys_{kt}}}$ is a vector of the responsible physician’s network characteristics; $Volume_{{Phys}_{kt}}$ is the number of patients the responsible physician encountered throughout the year; and $Specialty_{{Phys}_{kt}}$ is the specialty of the physician who prescribed drugs to patient *k* at time interval *t*. The elements of the $Network_{{Phys}_{kt}}$ and $Volume_{{Phys}_{kt}}$ measures were divided by their standard deviations; hence, the estimated coefficients correspond to a one-standard-deviation change in the predictor. The specification of random effects, $\theta _{k}^{(v)} \sim Normal(0, \sigma _{v}^2)$ and $\gamma _{Phys_{kt}}^{(v)} \sim Normal(0, \tau _{v}^2)$, accounts for the clustering of observations within patients and within physicians, respectively.

#### Modeling opioid-focused transitions

Because the combination of opioids and benzodiazepines or sedative-hypnotics are well-recognized risky medication combinations (Cho et al. 2020; Dowell et al. 2016; Sun et al. 2017), the prescription state transitions from an opioids-only state, denoted by *O*, to a state involving the combination of *OB* (opioid/benzodiazepine), *OS* (opioid/sedative-hypnotic), or *OBS* (opioid/benzodiazepine/sedative-hypnotic) are of particular interest. We are interested in the odds of a patient transitioning from *O* to $\{OB, OS, OBS\}$ (risky transitions) versus to state *zero* (non-risky transition) and its association with patient and physician characteristics. Because there are only two possible outcomes of this transition, a mixed-effect logistic regression simplification of Eq. 3 was used for this analysis.

#### Modeling patient’s time to discontinuation of a risky prescription state

We are particularly interested in how long a patient remains in state *OBS* (concurrent opioid, benzodiazepine, and sedative-hypnotic prescriptions), the riskiest state, and its association with the physician’s position in the network. Because many observations of the time until a patient departs state *OBS* were censored, we modeled time in state *OBS* using a mixed-effect Cox proportional hazard model, censoring those observations in which the patient was still in state *OBS* at the end of follow-up. The model has a general form:

4 $$\begin{aligned} \begin{aligned} h_{k}(t) =&h_0(t)exp(\beta _0 + \beta _1 Patient_{k} + \beta _2 Network_{Phys_{kt}} + \beta _3 Volume_{Phys_{kt}} \\ &+ \beta _{4} Specialty_{Phys_{kt}} + \theta _{k} + \gamma _{Phys_{kt}}), \end{aligned} \end{aligned}$$

where $h_0(t)$ denotes the unspecified baseline hazard function and the predictors and distributions of the random effects are as for the model in Eq. 3.

## Results

The shared-patient network formed from all beneficiaries residing in Ohio in 2014 who had face-to-face physician-patient encounters consisted of 57,581 U.S. physicians, including 50,643 in the LCC, from which the physician centrality measures were computed. The Medicare Part D prescription drug events contained 270,621 patients who had received opioids, benzodiazepines, or sedative-hypnotics prescribed by 32,058 physicians out of the 50,643 in the LCC, defining the sample of patients and their physicians used for outcome analyses. See Table 1 for patient and physician characteristics.

**Table 1:** Characteristics of patients in the study cohort and network statistics of the prescribing physicians

Characteristic or Statistic	%
Patients in study cohort^1^ (N = 270,621)	
Age (median, IQR)	72 (68, 78)
Female	65.62
Race	
White	86.25
Black	8.64
Other	5.11
Prescribing physicians for patients in study cohort^2^ (n)	32,058
Network measures (median, IQR)	
Closeness centrality	0.27 (0.21, 0.34)
Betweenness centrality^3^	1.72 (0.00, 6.96)
Eigenvector centrality^4^	0.03 (0.00, 2.71)
Clustering coefficient	0.10 (0.00, 0.16)
Specialty	
Primary Care	40.47
Emergency Medicine	15.35
Neurology	2.54
Psychiatry	3.24
Other	38.40
Volume^5^ (median, IQR)	* (*, 80)

^1^ The study cohort includes beneficiaries meeting inclusion criteria and who have filled at least one prescription of opioids, benzodiazepines, or sedative-hypnotics in 2014 prescribed by physicians in the physician shared-patient network of Ohio

^2^ The physician shared-patient network of Ohio is constructed based on shared patients from Ohio-residing beneficiaries from 2014, and different centrality measures are computed to represent physicians’ network position from 2014. Here the largest connected component of the constructed physician network (n = 50,643) is used to ensure the network is fully connected for computing distance-based centrality measures

^3,4^ The betweenness centrality and eigenvector centrality shown here are rescaled by multiplying the original values by $10^5$ for the clarity of presentation

^5^ Volume is the total number of patients a physician encountered annually. The * values are suppressed to meet the Centers for Medicare and Medicaid Services rules regarding suppression of cell sizes under 11

### Descriptive statistics: patient prescription-state transition matrix

**Fig. 1:**
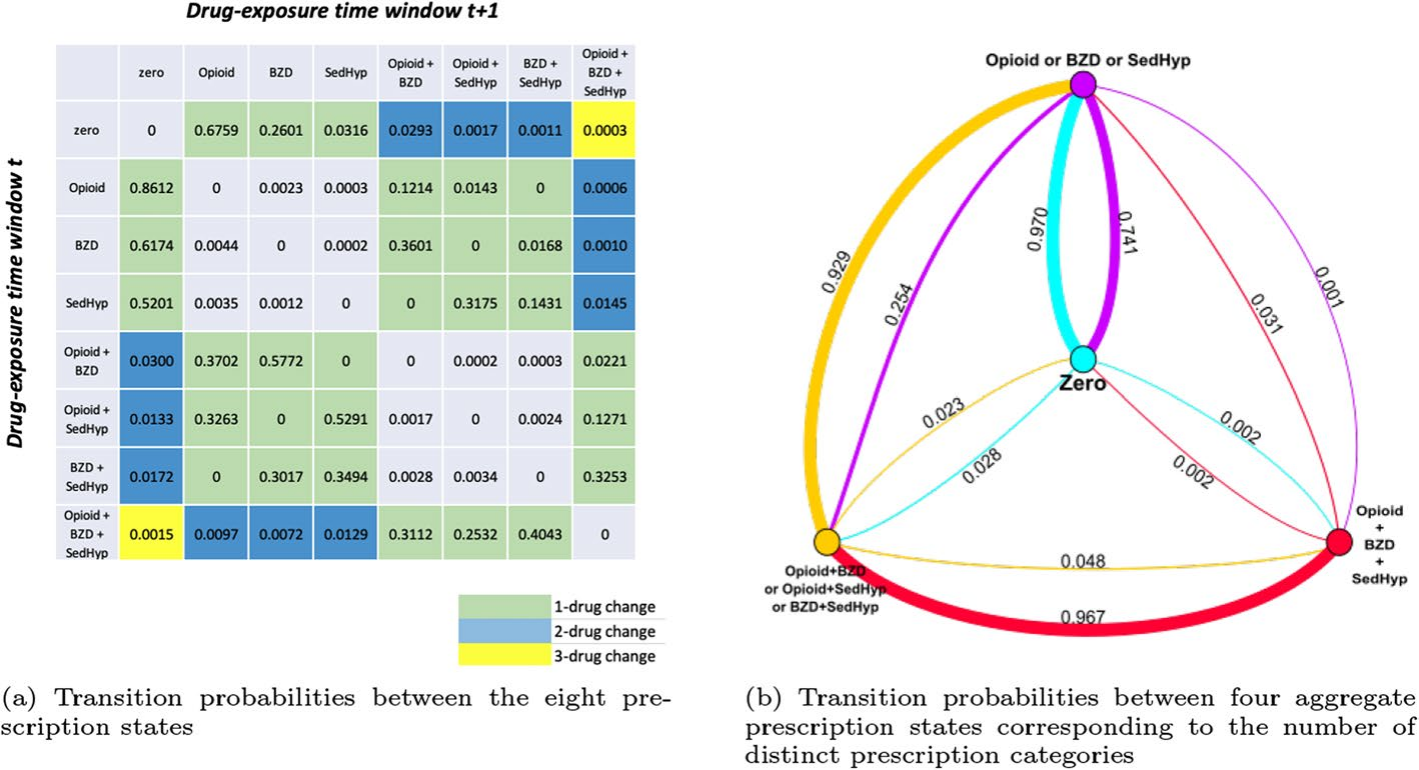
Prescription-state transition probabilities across all the patients in the study patient cohort. Panel **a** is an 8-prescription-state transition matrix across all the patients in the study cohort. Prescription states on the rows are the starting ("from") states, and prescription states on the columns are the next ("to") states. Different color scales correspond to 1, 2, or 3-drug changes respectively. Each cell represents the transition probability from the starting state to the next state. The probabilities may not sum to 1 across the rows due to rounding. Panel **b** displays the transition probabilities between the four states corresponding to the number of distinct prescriptions, represented by nodes in different colors. Zero means the patient is on no drugs in any of the three classes, Opioid or BZD or SedHyp means that they are on a drug (or drugs) in just one class, Opioid$+$BZD or Opioid$+$SedHyp or BZD$+$SedHyp means that they are on drugs in two classes and Opioid$+$BZD$+$SedHyp means that they are on drugs in all three classes. The proportions on the arcs between the classes are colored according to the sender node and reflect the proportion of times a patient who was in the sender drug state transitioned to the receiver drug state, with edge thickness reflecting the estimated transition probabilities. For example, 0.970 of the time, a patient transitioned from Zero to One drug class (Opioid or BZD or SedHyp) while the reverse occurred only 0.741 of the time. Self-edges of states of taking one distinct drug group or taking two overlapping drug groups are not shown; thus, the sum of the probabilities of edges in the same color may not equal 1. Abbreviations: BZD = benzodiazepine; SedHyp = non-benzodiazepine sedative-hypnotic

The 8 by 8 transition matrix summarizes the transition probabilities between the individual prescription states across all patients in the study cohort who received at least one drug in these three groups in 2014 (Fig. 1). By construction, all patients started from an initial state of receiving zero drugs in the three groups with each known to receive at least one drug across the three groups during 2014. Patients were more likely to make 1-drug group than 2-drug or 3-drug group transitions. It was also uncommon for patients to completely switch to another non-overlapping prescription state, such as from state *OB* (taking an opioid and a benzodiazepine) to state *S* (taking a sedative-hypnotic). Starting from state *zero*, among beneficiaries experiencing a transition, the proportion transitioning to state *O* or state *B* (only taking an opioid or a benzodiazepine) was 0.94. Physicians were more likely to prescribe benzodiazepines rather than sedative-hypnotics to patients in state *zero*, despite similar overall utilization of benzodiazepines and sedative-hypnotics. From state *O*, an opioids-only state, the proportion of transitions to state *zero* was 0.86. However, transitions to riskier prescription states were not uncommon. The proportion of transitions to state *OB*, taking an opioid and a benzodiazepine simultaneously (a risky combination), was 0.12. Similarly, from state *B*, a benzodiazepines-only state, patients generally returned to state *zero* (proportion 0.62) or added an opioid to transition to state *OB* (proportion 0.36). From state *S*, the sedative-hypnotics-only state, most patients discontinued the drug (proportion 0.52), while the proportion of times an opioid or a benzodiazepine was added on top of the sedative-hypnotic was 0.46. From state *OB*, the proportion of transitions to *O* or *B* was substantially higher than those to state *zero*. From state *OS*, patients commonly dropped one of the drugs they had been taking by their next new prescription. This “minimal-change” trend also occurred at state *BS*; patients were more likely to transfer to the adjacent *B*, *S*, and *OBS* states than return to state *zero* or to simultaneously discontinue the benzodiazepine or sedative-hypnotic and start on an opioid. This also applied when patients were in state *OBS*; they were more likely to stop filling prescriptions for one drug group and to transition to *OB*, *OS*, or *BS* than they were to return to state *zero*.

### Physician network position and patient number-of-drug-groups state transitions

To identify the physician network measures most associated with patient prescription state transitions, we first developed the best model to explain the data. Across the different models and the four measures of physician network centrality (node strength, closeness centrality, betweenness centrality, and eigenvector centrality) and physician clustering coefficient, we found that representing network position by physician closeness centrality and local clustering coefficient alone yielded the best fitting model. For parsimony, we only present results for this model. However, physician volume (number of patients encountered in 2014) was found to have a significant interaction with closeness centrality and better model fit (lower Akaike Information Coefficient (AIC) in the 2-drug prescribing transition models); see the Supplemental Appendix for this and other fitted models.

Table 2 shows the model coefficients (odds-ratios) for patients’ prescribing transitions. By setting the 1-drug prescribing transitions (from state *zero* to *O*, *B*, or *S*) as the reference level, the model parameters estimate the relative odds of 2-drug prescribing (transitions from state *zero* to *OB*, *OS*, or *BS*) and 3-drug prescribing (transitions from state *zero* to *OBS*). Older patients were less likely to experience 2-drug or 3-drug prescribing transitions ($OR_{2-drug}=0.957$, $p<0.001$;$OR_{3-drug}=0.928$, $p<0.001$), female patients were more likely to receive 2-drug prescribing ($OR=1.211$, $p<0.001$), and Black non-Hispanic patients were less likely to receive 2-drug prescribing ($OR=0.809$, $p<0.001$).

**Table 2:** Multinomial regression model estimates of the association between patients’ prescribing transitions and physicians’ network measures, adjusted for other covariates

	2-drug prescribing (State *zero* to states $\{OB, OS, BS\}$)		3-drug prescribing (State *zero* to state *OBS*)	
Predictor	Odds Ratio (95% CI)	*p*-value	Odds Ratio (95% CI)	*p*-value
Age	0.957 (0.955–0.960)	< 0.001	0.928 (0.905–0.951)	< 0.001
*Gender (Ref: Male)*				
Female	1.211 (1.168–1.256)	< 0.001	1.359 (0.912- 2.026)	0.132
*Race (Ref: White)*				
Black	0.809 (0.761–0.860)	< 0.001	0.900 (0.492–1.647)	0.732
Other	0.795 (0.727–0.869)	< 0.001	0.817 (0.331–2.014)	0.661
Closeness centrality	0.923 (0.907–0.939)	< 0.001	0.785 (0.657–0.938)	0.008
Clustering coefficient	1.025 (1.009–1.042)	0.002	0.939 (0.777–1.136)	0.519
Volume	1.034 (1.018–1.051)	< 0.001	1.005 (0.848–1.191)	0.953
*Specialty (Ref: Primary Care)*				
Neurology	0.522 (0.444–0.614)	< 0.001	0.693 (0.170–2.833)	0.610
Psychiatry	0.981 (0.891–1.080)	0.695	2.250 (1.185–4.270)	0.013
Emergency Medicine	0.506 (0.463–0.552)	< 0.001	0.194 (0.048–0.792)	0.022
Other	0.661 (0.633–0.690)	< 0.001	0.428 (0.251–0.730)	0.002

The multinomial prescribing transitions include 3 groups of transitions. 1-drug prescribing transitions are transitions from prescription state *zero* to states $\{O, B, S\}$ where O = opioid, B = benzodiazepine, S =non-benzodiazepine sedative-hypnotic. The 2-drug prescribing transitions are transitions from prescription state *zero* to states $\{OB, OS, BS\}$. The 3-drug prescribing transitions are transitions from prescription state *zero* to state *OBS*. The 1-drug prescribing transitions were set as the reference in the multinomial logistic model. Here volume is the physician’s annual volume of patients

Patients treated by physicians with a higher closeness centrality in the Ohio network were less likely to experience 2-drug or 3-drug prescribing; the association between closeness centrality and prescribing was stronger for transitions to three-drug groups than transitions to two-drug groups ($OR_{2-drug}=0.923$, $p<0.001$;$OR_{3-drug}=0.785$, $p=0.008$). Patients treated by physicians with a higher clustering coefficient or annual volume of patients were slightly more likely to experience a 2-drug prescribing transition ($OR_{Clust}=1.025$, $p=0.002$;$OR_{Vol}=1.034$, $p<0.001$). Compared to primary care physicians (PCPs), psychiatrists were more likely to be associated with 3-drug prescribing ($OR_{Psych}=2.250$, $p=0.013$), emergency medicine physicians were less likely to instigate a 2-drug or a 3-drug transition ($OR_{2-drug}=0.506$, $p<0.001$; $OR_{3-drug}=0.194$, $p=0.022$), and neurologists were associated with a lower likelihood of 2-drug prescribing ($OR=0.522$, $p<0.001$).

### Physician network position and patient opioid-focused prescription state transitions

A patient receiving care from a physician with a high clustering coefficient was more likely to add a benzodiazepine or sedative-hypnotic to the opioid they were taking ($OR = 1.030$, $p<0.001$, see Table 3). A higher patient-volume of a physician was associated with a slightly lower likelihood of such transitions ($OR = 0.990$, $p<0.001$). Patients treated by physicians with a higher closeness centrality were less likely to undergo such risky transitions ($OR = 0.986$ under a one-standard-deviation increase in their physician’s closeness centrality, $p<0.001$). Patients receiving an opioid prescription who were treated by a psychiatrist (versus a PCP) had a greater likelihood of adding a benzodiazepine or a sedative-hypnotic ($OR=4.248$, $p=0.008$). Finally, compared to PCPs, emergency medicine physicians had a lower likelihood of prescribing a benzodiazepine or a sedative-hypnotic to patients already receiving an opioid ($OR=0.360$, $p=0.012$).

**Table 3:** Mixed-effect logistic regression model estimates for patients’ prescription state transitions from state *O* to states *OB*, *OS*, or *OBS* (risky) versus from state *O* to state *zero* (non-risky), accounting for clustering of physicians

Predictor	Odds ratio (95% CI)	*p*-value
Age	1.005 (1.003–1.007)	< 0.001
*Gender (ref: male)*		
Female	0.862 (0.858–0.867)	< 0.001
*Race (ref: white)*		
Black	1.018 (1.013–1.023)	< 0.001
Other	0.748 (0.384–1.458)	0.393
Closeness centrality	0.986 (0.981–0.990)	< 0.001
Clustering coefficient	1.030 (1.025–1.035)	< 0.001
Volume	0.990 (0.986–0.995)	< 0.001
*Specialty (ref: primary care)*		
Neurology	0.739 (0.268–2.033)	0.558
Psychiatry	4.248 (1.450–12.443)	0.008
Emergency medicine	0.360 (0.163–0.796)	0.012
Other	0.577 (0.388–0.858)	0.007

State *O* is an opioids-only state, and states *OB*, *OS*, and *OBS* are states that involve the overlapping prescriptions of two drugs in the drug group pairs of opioids/benzodiazepines, opioids/sedative-hypnotics, or three drugs in the drug group combination of opioids/benzodiazepines/sedative-hypnotics. State *zero* is a clean-state free of drugs in the three groups. A random intercept for physicians was included in the model to account for their clustering. In this opioids-focused analysis, the ratio of the total number of transitions to the number of unique patients is 1.77, indicating that on average each patient made fewer than two transitions. Therefore, the clustering of patients is not considered in this analysis

### Physician network position and patient’s time to discontinuation of a risky prescription state

To gain insight into the factors affecting the end of a prescribing episode, we estimated the association of the network position of the physician who brought a patient into state *OBS* and the time that the patient remained in this riskiest state. Table 4 shows that a higher closeness centrality was associated with a higher likelihood of leaving state *OBS* (*hazard ratio (HR)* = 1.044, $95\%$ *CI*: [1.001, 1.089],* p* = 0.047). By adding random effects of patients and physicians to account for clustering, the effect of physician closeness centrality on patient’s time to discontinuation from state *OBS* was attenuated and estimated with less precision (*HR* = 1.018, $95\%$ *CI*: [0.923, 1.123],* p* = 0.726). In both of the models with and without the random effects of patients and physicians, patients who encountered prescribers with a higher clustering coefficient or higher annual volume of patients exited state *OBS* more rapidly ($HR_{Clust}$ = 1.081, $95\%$ *CI*: [1.002, 1.165],* p* = 0.043; $HR_{Vol}$ = 1.109, $95\%$ *CI*: [1.006, 1.223],* p* = 0.038). However, patients whose prescriber was a psychiatrist compared to a PCP took longer to exit state *OBS* ($HR_{Psych}$ = 0.663, $95\%$ *CI*: [0.507, 0.867],* p* = 0.003), while those who had seen an emergency medicine physician exited more quickly ($HR_{Emerg}$ = 8.276, $95\%$ CI$: [5.775, 11.860], p < 0.001$).

**Table 4:** Cox Proportional Hazards model estimates of the association between patients’ time to discontinuation of state *OBS* and network measures of prescribing physicians, adjusted for other covariates

	Model 1		Model 2	
Predictor	Hazard ratio (95% CI)	*p*-value	Hazard ratio (95% CI)	*p*-value
Age	1.014 (1.010–1.018)	< 0.001	1.029 (1.019–1.039)	< 0.001
*Gender (ref: male)*				
Female	1.068 (0.985–1.157)	0.110	1.143 (0.944–1.384)	0.171
*Race (Ref: White)*				
Black	1.116 (0.987–1.263)	0.081	1.282 (0.950–1.730)	0.105
Other	1.078 (0.881–1.319)	0.464	1.163 (0.744–1.815)	0.509
Closeness centrality	1.044 (1.001–1.089)	0.047	1.018 (0.923–1.123)	0.726
Clustering coefficient	1.044 (1.010–1.080)	0.011	1.081 (1.002–1.165)	0.043
Volume	1.056 (1.013–1.101)	0.011	1.109 (1.006–1.223)	0.038
*Specialty (ref: primary care)*				
Neurology	0.828 (0.625–1.096)	0.186	0.877 (0.490–1.569)	0.670
Psychiatry	0.706 (0.619–0.805)	< 0.001	0.663 (0.507–0.867)	0.003
Emergency medicine	3.234 (2.656–3.939)	< 0.001	8.276 (5.775–11.860)	< 0.001
Other	1.138 (1.036–1.249)	0.007	1.487 (1.218–1.815)	< 0.001

Model 1 is a Cox proportional hazards regression model. Model 2 is a mixed-effect (or group-frailty) Cox proportional hazards model using random effects to account for the clustering of patients and physicians. We modeled patients’ time to their discontinuation of state *OBS*, and events were censored if patients had not discontinued state *OBS* by the end of the study. State *OBS* is a prescription state of concurrently taking an opioid, a benzodiazepine, and a sedative-hypnotic

## Discussion

We found several physician-level factors including structural features of their position in a shared-patient network were associated with a patient’s risky drug receipt. Greater physician network prominence as measured by closeness centrality was associated with a modestly safer prescribing practice, including a lower likelihood of prescribing multiple risky drugs, a lower likelihood of prescribing drugs leading to risky combinations, and prescribing risky drug combinations for shorter times. Physician specialty was understandably associated with patient risky drug receipt. Compared to PCPs, psychiatrists were more likely to be involved in the risky prescribing of opioids, benzodiazepines, and sedative-hypnotics and their patients stayed in the riskiest state (overlapping prescriptions of all three drug groups) for longer. Emergency medicine physicians were less likely to conduct risky prescribing and their patients experienced shorter periods of time in risky prescription states compared to patients served by PCP prescribers (Jeffery et al. 2018).

Our finding that physicians in structurally important network positions in terms of closeness centrality exhibited safer prescribing practices, conveys a positive message. This suggests that physicians with greater influence and opportunity to disseminate information exercise more caution by limiting patient exposure to problematic combinations of drugs. Because closeness centrality is associated with the intensity of sharing patients (with more shared patients leading to a shorter distance and likely greater closeness for the associated physicians), our findings may also reflect the importance of collaboration between physicians in battling risky prescribing.

Our findings corroborate those of previous studies, in which increased patient-sharing among pairs or groups of physicians was associated with guideline-recommended prescribing and a lower likelihood of risky drug prescribing (Fattore et al. 2009; Arnold et al. 2021; Ong et al. 2016). It may be helpful to develop interventions designed to increase the connectivity of peripheral physicians (with low closeness centrality) whose prescribing was less guideline-adherent. For example, a peer-oversight or mentoring program, or a program that encouraged greater consultation with other physicians on challenging cases, could be targeted to physicians who share fewer patients with other physicians to help them to engage with other physicians more when prescribing. In addition, the increased risk observed for females of *zero* to 2-drug transitions is consistent with results observed in multiple countries (Correia et al. 2019; Sánchez-Valle et al. 2024).

The inconsistent findings with respect to the local clustering coefficient and the annual volume of patients on their patients’ likelihood of risky drug receipt may be due to these terms being associated with multiple phenomena. A physician’s local clustering coefficient quantifies the intensity of connections among their own peers, which in some instances may reflect collaboration cohesiveness. However, our finding that physicians with a higher local clustering coefficient had a slightly higher likelihood of prescribing risky combinations of drugs, may indicate that physicians who care for the most severe patients may collaborate with or seek advice from other physicians more often. Previous studies also suggest that complex and intense drug combinations can be necessary for certain patients (Viola et al. 2004). Moreover, we found that among patients that experienced concurrent use of risky drugs, patients served by physicians with a higher (versus lower) local clustering coefficient have shorter duration times in these states. This may reflect physician caution and judgment when risky prescribing is inevitable; recognizing the risk, they try to minimize the harm by prescribing for a short time period.

Likewise, the mixed impact of physicians’ annual patient volume on their patients’ risky drug receipts may also reflect multiple phenomena and heterogeneity between patients. The volume-outcomes literature in health services research has documented that a higher physician volume of patients is generally associated with better patient outcomes (Halm et al. 2002; Joynt et al. 2013; Tu et al. 2001; Landon et al. 2010; O’Malley et al. 2011). Higher-volume physicians may be more experienced and better at determining drug regimens suitable for a patient’s needs. However, they may also attract more serious cases in ways not reflected in the data, which warrant greater collaboration but also riskier prescribing.

Patients’ receipt of risky drugs and their combinations varied by the specialty of the physician who cared for them. Compared to PCPs, psychiatrists were more likely to prescribe multiple drugs, to prescribe the riskiest combinations, and to prescribe drugs leading to risky combinations for a longer time. These findings may reflect that psychiatrists commonly handle complex situations such as patients who are unresponsive to standard (or recommended) treatment that needs multiple prescriptions (Kukreja et al. 2013). When such medical complexity is not measured in the data and thus omitted from statistical models, the association of psychiatrist prescribers with a greater likelihood of risky prescribing could reflect complex medical needs rather than inappropriate medical practice. In contrast, risky prescribing behavior among PCPs might be more problematic and may explain the prevalence of drug-related adverse events in primary care (Gandhi et al. 2003). In addition, PCPs, as the common first point of contact when patients reach out for medical treatment, are in a crucial position to avert patients from unnecessary polypharmacy, and thus might be the prime target for an intervention aimed at reducing unnecessary polypharmacy. In general, our findings suggest the need for coordinated care from different personnel to mitigate the harm of risky prescriptions.

A general limitation of our study is that the data are observational, which as alluded to above, allows for multiple causal explanations for the observed associations. Another limitation is that we observe the prescriptions filled by a patient and use this as a surrogate of whether the patient was truly taking the drugs. We are also limited in our ability to balance the good with the bad. While we observe prescriptions, we do not observe decisions not to fill. Therefore, it was not possible to model the likelihood that a physician reduces polypharmacy as a function of the structural characteristics of their network position, their specialty and other characteristics. However, to gain some insight into deprescribing, we modeled the time a patient remained in the riskiest prescription state in terms of the network and the individual characteristics of the physician responsible for entering them into that state. With the data being from 2014 and thus somewhat dated, they may not reflect current risky prescribing patterns. In particular, the landscape for prescribing these drugs may have changed over time. However, because we do not analyze individual drugs and combinations of drugs but rather focus on drug groups, we believe it is highly unlikely for the relevance of our results to have meaningfully diminished. Another limitation is that we only analyzed data from the US state of Ohio. However, as noted in Section 2.1, our analyses are not intended to be representative of the entire US. In contrast, the analyses we performed on Ohio are intended to be symbolic of analyses that may be performed across many different applications and scales of data.

The key point is that, in the form of our methodology, we now have an approach that allows us to understand and quantify the role of shared patient prescribing networks in risky prescribing. We can apply our methodology to any given application to identify, for example, potentially influential risky prescribers (those with high network centrality and high risky prescribing) to target with interventions. If successful, such interventions could be both efficient (by only needing to target a few high impact souls) and effective (if the intervention works). This, of course, is what the Pharmaceutical industry has been doing for decades– identifying and imposing lavishing gifts on the most connected, respected, and influential prescribers to advance use of its products (Schwartz and Woloshin 2019). We propose the opposite, using our method to identify influencers, in this case high-impact-Rx influencers perhaps in the form of an educational intervention that advances prescribing quality and safety.

There is a substantial amount of future research that follows from this paper. For example, if prescriber demographic characteristics such as age (or years since graduation) had been available we could have examined whether risky prescribing was more prone to occur in one demographic of provider versus another and accounted for any confounding effect of prescriber age (e.g., if prescribers of different ages have both different prescribing tendencies and network positions). Because such variables were not available, this remains a topic for future research. Another avenue for future research is to use recently developed methods for constructing directed shared-patient networks (O’Malley et al. 2023, 2025) to allow directed variants of all of the network measures (e.g., in-degree and out-degree versus undirected degree and analogously for other network prominence measures) to be used as network-based predictors in models like those estimated herein.

## Conclusion

For the three drug groups we studied, this work improves our understanding of physicians’ involvement in risky prescribing and demonstrates the potential of network analysis to identify physicians that might be best to intervene on to reduce risky or unnecessary prescribing. By using novel data constructs and network science methods, we studied risky prescribing through a new lens and obtained novel findings. Furthermore, the new methods that we have introduced provide a general framework for quantifying patients’ receipt of risky drug combinations and the physician-related factors associated with prescription state transitions in any area of medicine. The methods can also be generalized to studies involving more groups of drugs.

## Additional file

Supplementary file 1 (pdf 760 KB)

## Data Availability

The data used for the empirical analyses reported in this paper contain patient-identifiable information and so cannot be made available. However, additional details about the construction of the data and the various summary measures involved in the descriptive and model-based statistical analyses are provided at the GitHub site of the paper: https://github.com/kiwijomalley/PhysicianNetworkRiskyPrescribing.
