# Supplementary material for: The association of prescriber prominence in a shared-patient physician network with their patients receipt of and transitions between risky drug combinations
Source: Appl Netw Sci. 2025 Jul 21;10(1):34. doi: 10.1007/s41109-025-00721-y (PMC12279612; doi:10.1007/s41109-025-00721-y)
Supplement: Supplementary file 1 — (pdf 760 KB) [file 41109_2025_721_MOESM1_ESM.pdf]

The association of prescriber prominence in a shared-patient physician network with their patients receipt of and transitions between risky drug combinations

SUPPLEMENTAL ONLINE APPENDIX

Section A: Additional details of network and analytic dataset construction

Cohort definition and study workflow

Figure A1 is a diagram of the cohort definition and study workflow. We built a shared-patient network from Medicare Parts A and B claims of beneficiaries residing in Ohio in 2014 receiving all types of medical care (including non-risky drugs and other medical procedures) consisting of 57,581 physicians of which 50,643 were in the largest connected component (LCC), and from which different physician centrality measures were computed. Furthermore, a total of 270,621 patients were included from the Medicare Part D claims who had been prescribed opioids, BZDs, or SedHyps by 32,058 physicians in the LCC of network in 2014, forming the sample of patients and physicians who prescribed to them in the outcome analyses. Specifically, this sample included 208,425 patients who had experienced transitions from state zero to states {O, B, S} (filling for one drug), or to states {OB, OS, BS} (filling for two drugs), or to state OBS (filling for three drugs) in the analysis of drug state transitions based on volume. For the analysis of opioid-focused transitions, 13,546 patients were included who had experienced transitions from state O to states {OB, OS, OBS} (risky transitions) versus to state zero (non-risky transition). Lastly, for the analysis of patient's time to discontinuation of a risky drug state, 1,786 patients were included who had entered the risky drug state OBS of taking an opioid, a BZD, and a SedHyp concurrently during the study period. Observations were censored if the patient was still in state OBS at the end of follow-up.

Modeling patient risky drug state

Several studies have quantified a patient's receipt of possibly problematic drug combinations, which focused on the incidence of multi-drug exposure.[1,2] To build on this knowledge, an important step is to quantify patient's prescription receipt in terms of drug states which account for both the number and class of the drugs. This allows deeper understanding of the initiation and discontinuation of prescriptions, along with the dynamics of the receipt of risky drug combinations. We were more interested in the initialization of a prescription and the prescriber who started the prescription. Therefore, we focused on new prescription fills instead of refills, and the initial prescriber of a long prescription chain. Specifically, if the gap between a preceding and a subsequent prescription fill is shorter than 20% of the fill length of the preceding fill, we treated the subsequent fill as a refill of its precedent. Furthermore, if the subsequent fill was linked to a different prescriber, we still merged this fill to its preceding fill and attributed it to the previous prescriber. Then we utilized a counting process to generate discrete and non-overlapping time windows in which a new data entry was triggered when a prescription fill started or ended.

Figure A1. Cohort definition and study workflow.

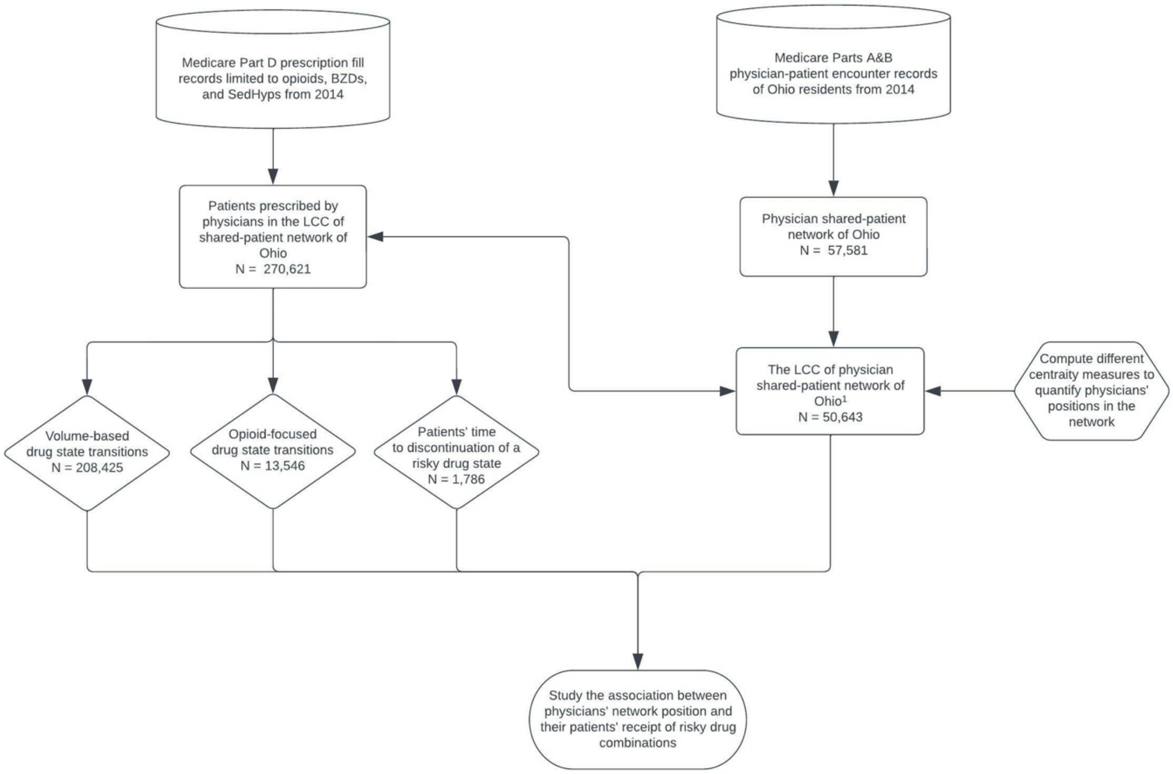

[1] The largest connected component (LCC) of the physician shared-patient network was utilized because some of the centrality measures are distance-based, e.g., closeness centrality and betweenness centrality, and isolates in the network will have infinity distance to other nodes as will nodes in one component of the network to nodes in another component. Therefore, it is a common practice that the LCC of network is used to ensure the nodes are fully connected and reasonable distances can be obtained.

Next, we utilized a state space framework to quantify the dynamic patterns in a patient's receipt of risky drugs and their combinations. Based on the number and class of drugs a patient was taking during each of the discrete and non-overlapping time windows generated by the counting process, the patient has a state space S including eight possible states,

$$S = \{zero, O, B, S, OB, OS, BS, OBS\}$$

where state zero corresponds to taking no drugs, and other states indicate the class or the combination of drug classes the patient was taking ("O" for opioids, "B" for BZDs, and "S" for SedHyps). **Figure A2** shows the workflow of modeling patient drug states.

Figure A2. Workflow of modeling patient drug states.

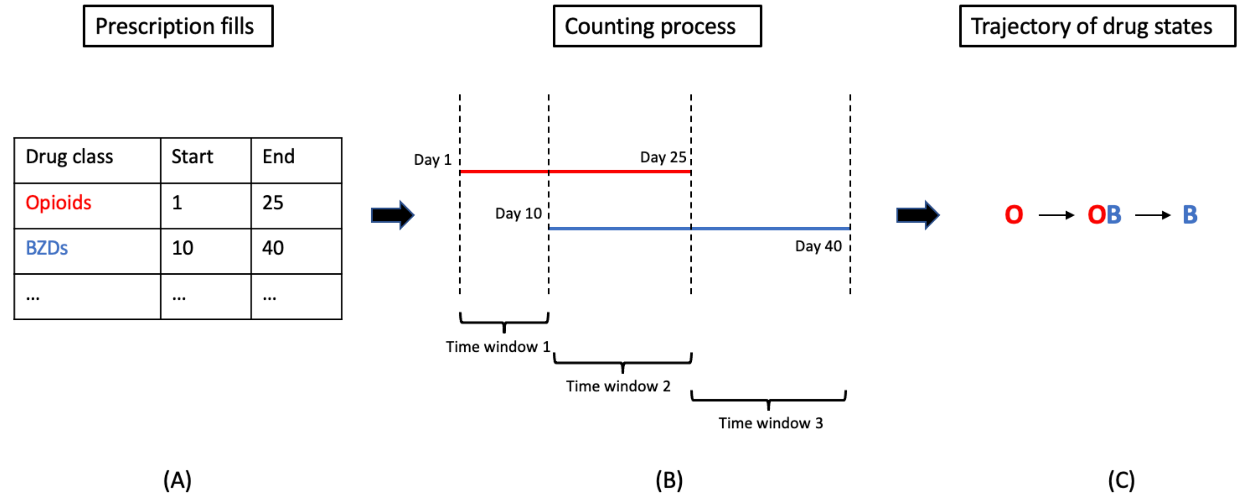

Note: Panel (A) shows a made-up example of patient's sequence of prescription fills with their corresponding drug class, the start and end of the fill. Panel (B) shows the counting process to split the sequence of prescription fills into discrete time windows which reflect the initialization and the discontinuation of a prescription fill. The red line indicates the prescription fill length of the opioid in panel (A), and the blue line indicates BZD fill length. Panel (C) shows the corresponding drug state during each time window in panel (B) and the transition between them, forming a trajectory of drug states across time. "O" stands for filling for an opioid, "B" stands for filling for a BZD, and "OB" stands for filling for an opioid and a BZD concurrently.

For each patient, we obtained a trajectory of drug states from the time sequence of their prescription fills. Then we modeled a patient's trajectory of drugs states as states along a Markov chain. Suppose D_t is a patient's drug state during their ordered time window t, and s is the value that D_t can take. We assumed the Markov property that the transition probability to a certain drug state was only dependent on the most recent former state regardless of the earlier states. Hence,

$$P(D_{t+1} = s \mid D_t = s_t, D_{t-1} = s_{t-1,} \dots, D_0 = s_0) = P(D_{t+1} = s \mid D_t = s_t)$$

Then we estimated an 8-by-8 transition matrix by summarizing the drug state trajectories across all patients, where each cell is the conditional probability of reaching another drug state in the next step starting from a certain drug state. Note that this 8-by-8 transition matrix does not assume the Markov property but estimating it does. As described in the section on Modeling Drug State Transitions Resulting in Increased Risky Combinations in the main text, based on the number of distinct drug class involved, the eight drug states can be further aggregated into four categories corresponding to taking 0, 1, 2, and 3 distinct classes of drugs. Category 1 is state zero, category II includes states {O, B, S}, category III includes states {OB, OS, BS}, and category IV is state OBS. **Figure A3** is a visualization of the eight drug-state transitions where each node represents one of the eight drug states.

Figure A3. Visualization of drug-state transition probabilities across all the patients in study cohort.

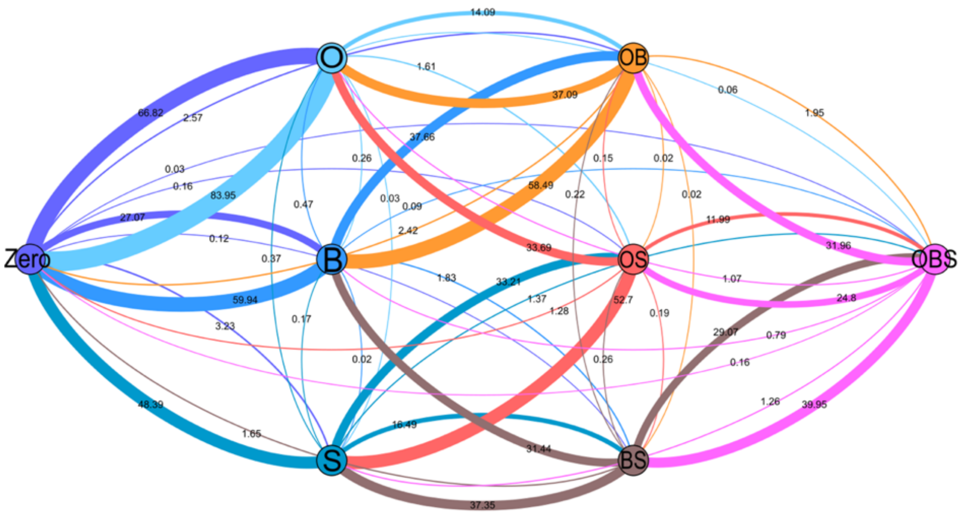

Note: A visualization of 8 drug-state transitions. The nodes represent 8 different drug states, and edge weights and thickness reflect the percentages of transitions from a given node. Node “zero” corresponds to the drug state of taking 0 drug in the three classes, nodes “O, B, S” represent the drug states that correspond to taking one drug either in the classes of opioids, BZDs, or SedHyps, node “OB, OS, BS” are drugs states corresponding to taking two drugs that are a combination of opioids/BZDs, opioids/SedHyps, or BZDs/SedHyps, and node “OBS” is the drug state of taking three drugs that is a combination of opioids, BZDs, and SedHyps. The nodes that emanate clockwise edges are the source nodes (states), and the nodes that receive clockwise edges are the target nodes (states). Edges in the same color of the nodes indicate the transitions originated from those nodes. The sum of the percentages of transitions from a node may not equal to 1 due to rounding.

Section B: Network position summary measures

Physician’s degree/strength centrality

Degree is the number of connections a node possesses in the network. For a binary network, node degree is given by,

$$k_i = \sum_j a_{ij}$$

The counterpart of degree in a weighted network is known as the node strength,[3] which is given by,

$$s_i = \sum_{j=1}^{N} w_{ij}$$

where a_{ij} is the ij’th element of the adjacency matrix, and w_{ij} is the edge weight linking nodes i and j.

Physician’s closeness centrality

Closeness centrality is a centrality measure based on geodesic distance measured by the inverse sum of geodesic distances from a certain node to all the other nodes in the network. It quantifies how far on average a node is to other nodes. Nodes with higher closeness centrality are considered to have higher efficiency in spreading information; they are considered “closer”

to other nodes in the network. For a binary network, the closeness centrality of a node *i* is defined as

$$C_c(i) = \frac{1}{\sum_j^N d(i,j)}$$

where *N* is the number of total nodes in the network, and $d(i,j)$ is the geodesic distance from node *i* to *j*. If the network consists of multiple components, the largest connected component is typically used to calculate centrality measures based on distance between node pairs. In this case, *N* is the number of nodes in the largest connected component. Closeness centrality can also be extended to weighted networks, where it is measured by the inverse of edge weight based on Dijkstra's shortest path algorithm.[4–6] The weighted geodesic distance (the path of minimum cost) from node *i* to *j* can then be defined as,

$$d^w(i,j) = \min\left(\frac{1}{w_{ih}} + \cdots + \frac{1}{w_{hj}}\right)$$

which is the minimum of the sum of the inverse weights from node *i* to *j*. Therefore, weighted closeness centrality is given by,

$$C_c^w(i) = \frac{1}{\sum_j^N d^w(i,j)}$$

Normalized closeness centrality was obtained by multiplying the raw closeness by $N - 1$.

Physician's betweenness centrality

Betweenness centrality measures the proportion of geodesic paths between pairs ("dyads") of physicians through the network that are intersected by the focal node. It reflects the node's capability to be privy to information flow.[7] Betweenness centrality can be extended to weighted networks.[8] For a weighted and undirected network, it is given,

$$C_b^w(i) = \frac{\sum_{i \neq j \neq k} \sigma_{jk}(i)/\sigma_{jk}}{(N-1)(N-2)/2}$$

where $\sigma_{jk}(i)$ is the sum of weights of the weighted geodesic paths between nodes *j* and *k* that pass-through node *i*, and σ_{jk} is the sum of weights of all the weighted geodesic paths between nodes *j* and *k*.

Physician's eigenvector centrality

Eigenvector centrality measures the influence of a node in a network in terms of the centrality of nodes to which it is connected. It accounts for not only the number of ties a node possesses, but also the importance, quality, or influence of the node's neighbors. Connections to nodes with higher influence are weighed more than those with lower influence. For a binary network, suppose *A* is the adjacency matrix, then the eigenvector centrality of node *i* is the ith element of vector C_e that satisfies the following equation,

$$AC_e = \lambda C_e$$

where C_e is an eigenvector of the adjacency matrix *A* with the eigenvalue of λ. Among the many eigenvectors that satisfy the above equation, C_e is the principal (leading) eigenvector associated with the principal eigenvalue; the Perron–Frobenius theorem indicates that the principal eigenvalue and the corresponding eigenvector can ensure non-negative centrality values.[9] This can be generalized to a weighted network, using edge weights in place of adjacency indicators.[10] Letting *W* denote the weighted adjacency matrix, the eigenvector centrality of node *i* is the ith element of the leading eigenvector C_e^w such that,

$$WC_e^w = \lambda C_e^w$$

and λ is the largest eigenvalue.

Physician's local clustering coefficient

Local clustering coefficient is a measure of nodes' tendency to cluster together and how often a node's neighbors are connected.[11] Nodes with higher clustering coefficient in a physician network indicate that they are more tightly connected with the physicians in their local clique, which might indicate more collaboration and cohesiveness. For a binary network, the local clustering coefficient of a node i is the ratio of the number of closed triads involving node i to the number of either open or closed triads that involve node i. It is computed as,

$$Clust_i = \frac{t_i}{k_i(k_i - 1)}$$

where t_i is the number of closed triads that involve node i, k_i is the degree of node i, $k_i = \sum_j a_{ij}$, and a_{ij} is the ij'th element of the adjacency matrix. The clustering coefficient can also be generalized to weighted networks; such as by the expression given in Barrat,[3]

$$Clust_i^w = \frac{1}{s_i(k_i - 1)} \sum_{j,h} \frac{w_{ij} + w_{ih}}{2} a_{ij} a_{ih} a_{jh}$$

where $s_i = \sum_j a_{ij} w_{ij}$ is the strength of node i and w_{ij} is the edge weight linking nodes i and j. Besides the number of closed triads among node i's neighbors, this extension to weighted networks accounts for the connection weights relative to the focal node's strength. The equation reduces to the binary version for binary networks (all the non-zero edge weights equal 1). The network measures were computed using the igraph package in R.[12]

Section C: Additional statistical models and results of analyses

Fitted models for other network measures

We had an explanatory but parsimonious process to determine the best model for the analysis. Although network centrality measures were theorized to reflect physician's structural importance in the network and can be associated with their practice in patient care, we did not know which network centrality measure was the most predictive. **Table C1** shows the correlation among different physician network measures. To infer the most influential physician network measures on patient's drug state transitions, we utilized manual iterative model-building to determine the appropriate set of network predictors. We started with the full model with all the network measures including node strength, closeness centrality, betweenness centrality, eigenvector centrality, local clustering coefficient, patient demographic factors and physicians' specialty and annual volume of patients. We also tested different combinations of the network measures by considering closeness centrality, betweenness centrality, and eigenvector centrality, along with clustering coefficient and physician volume. The reason why we formulate this type of combination is that these different measures reflect different aspects of a physician's role in the network. Centrality measures correspond to physician's structural importance, local clustering coefficient represents physician's collaboration and cohesiveness in their local cluster, and physician volume indicates their practice size.

We found that physician betweenness centrality and eigenvector centrality were not statistically significant predictors of the outcomes of interest. Further, closeness centrality and local clustering coefficient were the optimal combination of network measures in terms of model

fit that both avoided collinearity concerns and appeared free of spurious effects. Akaike's Information Criterion (AIC) was used to assess the model fit, and pointed to the model with closeness centrality and local clustering coefficient as the optimal specification across the models of the many transitions in patient polypharmacy status. The consistency of the results for closeness centrality and local clustering coefficient across the various models provides additional evidence of their overall importance than that captured in the confidence intervals and p-values for individual models.

Table C1. Pearson's correlation coefficients among different physician-level predictors.

	Strength	Betweenness centrality	Closeness centrality	Eigenvector centrality	Clustering coefficient	Volume
Strength						
Betweenness centrality	0.059					
Closeness centrality	0.667	0.054				
Eigenvector centrality	0.450	0.004	0.140			
Clustering coefficient	-0.059	-0.002	-0.043	-0.023		
Volume	0.443	0.005	0.201	0.263	-0.091	

Effect of physician closeness centrality on patient's drug receipt modified by physician volume

Physicians with higher volume of patients can face more complicated patient conditions and this may modify the association between their centrality in the network and their patients' risky drug receipts and discontinuation of risky drug states. Therefore, we also investigated whether these effects were modified by physician's annual volume of patients. Prior work has found that the peer effect of a hospital adopting implantable cardioverter defibrillators was modified by the ego hospital's network strength - the number of patients it shared with hospitals.[13] In our study, we theorized that physicians with larger practice size may treat more complicated conditions more demanding of multiple prescriptions, which may affect their prescribing decision even for the most central physicians. We found a statistically significant interaction between physician closeness centrality and volume with marginally improved model fit for patient's 2-drug prescribing transitions. Because adding this interaction led to worse model fit for the other two outcomes of interest, for easy comparability across models we excluded interactions for the analyses of time-to-discontinuation of a risky drug state and the opioid-focused transitions. **Table C2** shows the model estimates with the interaction term of closeness centrality and volume, and **Figure C1** is the plot showing the effect of physician closeness centrality on patient's 2-drug prescribing transitions modified by physician volume. We found

that higher physician closeness centrality was associated with patient's lower likelihood of experiencing 2-drug prescribing transitions compared to 1-drug transitions, though this effect was dampened by physician volume. According to the 95th percentile of volume on **Figure C1**, even with this modified effect, central physicians with the greatest volume of patients were still associated with lower likelihood of 2-drug prescribing.

Table C2. Model estimates of association between patients' 2-drug prescribing transitions and physicians' network measures with effect modification by physician volume, adjusted for other covariates.

	Estimate	Std. Error	p-value
Age	-0.044	0.001	< 0.001
Gender (Ref: Male)			
Female	0.191	0.019	< 0.001
Race (Ref: White)			
Black	-0.216	0.031	< 0.001
Other	-0.232	0.045	< 0.001
Closeness centrality	-0.084	0.009	< 0.001
Clustering coefficient	0.026	0.008	0.002
Volume	0.036	0.008	< 0.001
Closeness centrality*Volume	0.025	0.007	< 0.001
Specialty (Ref: Primary care)			
Neurology	-0.651	0.083	< 0.001
Psychiatry	-0.022	0.049	0.656
Emergency Medicine	-0.683	0.045	< 0.001
Other	-0.410	0.022	< 0.001

Note: The effect of physician closeness centrality on patients' 2-drug prescribing is given by -0.084 + 0.025×volume.

Figure C1. The effect of physician closeness centrality on patients' 2-drug prescribing.

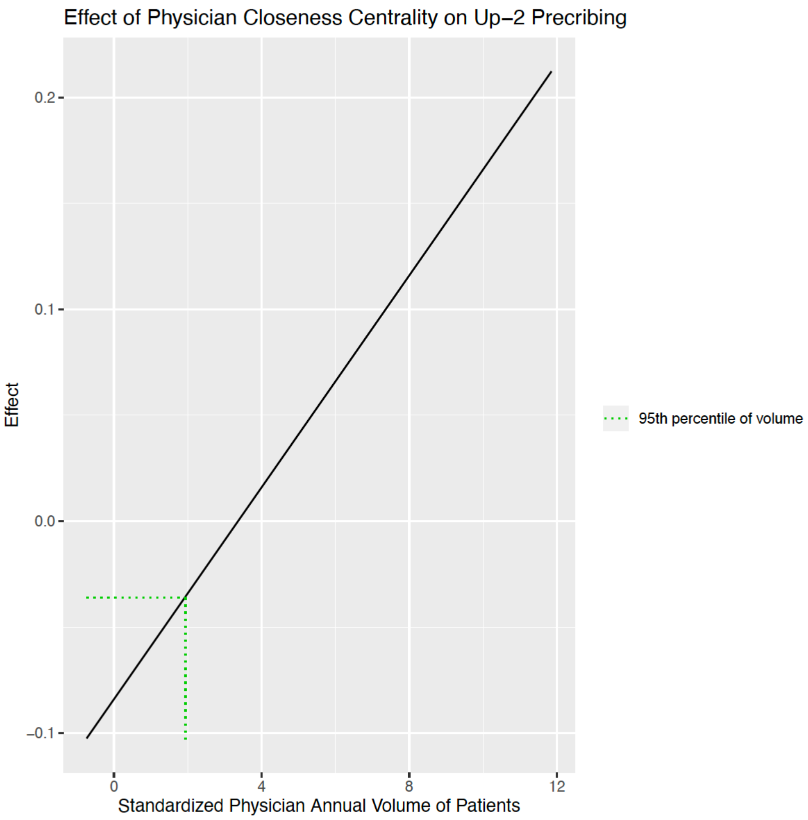

Note: This plot shows the log-odds of patient's experiencing 2-drug (up-2) prescribing compared to 1-drug prescribing with their prescribing physician's closeness centrality, modified by physician's annual volume of patients. The effect of physician's closeness centrality on patient's 2-drug prescribing transition is a function of physician volume. Here the physician annual volume of patients has been standardized by subtracting the mean and dividing by the standard deviation. The green dashed line indicates the 95th percentile of physician volume.

References

1. Sutherland JJ, Daly TM, Liu X, Goldstein K, Johnston JA, Ryan TP. Co-Prescription Trends in a Large Cohort of Subjects Predict Substantial Drug-Drug Interactions. *PLOS ONE*. 2015;10(3):e0118991.

2. Quinn KJ, Shah NH. A dataset quantifying polypharmacy in the United States. *Sci Data*. 2017;4(1):170167.

3. Barrat A, Barthélemy M, Pastor-Satorras R, Vespignani A. The architecture of complex weighted networks. *PNAS*. 2004;101(11):3747-3752.

4. Dijkstra EW. A note on two problems in connexion with graphs. *Numer Math*. 1959;1(1):269-271.

5. Newman ME. Scientific collaboration networks. II. Shortest paths, weighted networks, and centrality. *Phys Rev E*. 2001;64(1):016132.

6. Opsahl T, Agneessens, F., Skvoretz, J. Node centrality in weighted networks: Generalizing degree and shortest paths. *Soc Netw*. 2010;32(3):245-251.

7. Freeman LC. Centrality in social networks conceptual clarification. *Soc Netw*. 1978;1(3):215-239.

8. Brandes U. A faster algorithm for betweenness centrality. *J Math Sociol*. 2001;25(2):163-177.

9. Newman ME. The mathematics of networks. *New Palgrave Encycl Econ*. 2008;2(2008):1-12.

10. Newman ME. Analysis of weighted networks. *Phys Rev E*. 2004;70(5):056131.

11. Latapy M, Magnien C, Vecchio ND. Basic notions for the analysis of large two-mode networks. *Soc Netw*. 2008;30(1):31-48.

12. Csardi G, Nepusz T. The igraph software package for complex network research. *InterJournal Complex Syst*. 2006;1695(5):1-9.

13. O'Malley AJ, Moen EL, Bynum JP, Austin AM, Skinner JS. Modeling peer effect modification by network strength: The diffusion of implantable cardioverter defibrillators in the US hospital network. *Stat Med*. 2020;39(8):1125-1144.
